# Risk factors for liposomal bupivacaine resistance after total hip or knee arthroplasties: a retrospective observational cohort in 237 patients

**DOI:** 10.1186/s13037-020-0230-4

**Published:** 2020-01-30

**Authors:** Scott Buzin, Arianna L. Gianakos, Deborah Li, Anthony Viola, Sherif Elkattawy, David M. Keller, Richard S. Yoon, Frank A. Liporace

**Affiliations:** 1 Division of Orthopaedic Trauma & Adult Reconstruction, Department of Orthopaedic Surgery, Jersey City Medical Center – RWJBarnabas Health, 377 Jersey Ave, Suite 280A, Jersey City, NJ 07302 USA; 2 University of Miami Miller School of Medicine, 1600 NW 10th Ave #1140, Miami, FL 33136 USA; 3 Philadelphia College of Osteopathic Medicine, 4190 City Ave, Suite 409, Philadelphia, PA 19131 USA

**Keywords:** Liposomal bupivacaine, Total hip arthroplasty, Total knee arthroplasty, Pain management

## Abstract

**Purpose:**

Liposomal bupivacaine demonstrated promise decreasing postoperative pain in total hip and total knee arthroplasty (THA/TKA). Some randomized trials have shown non-superior results; however, confounding variables were not accounted for in such analyses. This study attempts to determine risk factors associated with failure of pain management in patients receiving liposomal bupivacaine.

**Methods:**

Postoperative pain scores were collected following primary or revision arthroplasties between January 2016 and December 2017. Retrospective analysis of institutional total joint quality and outcomes registry was screened and patients undergoing primary or revision arthroplasties who completed a multi-modal pain management including liposomal bupivacaine were included in the study. Patients with a history of infection/deviated from the institutional pain management protocol were excluded.

**Results:**

A total of 237 patients were included for analysis. Younger patients less than 64 years old had significantly higher pain scores between 0 and 12 h and > 24 h. Active smokers had significantly higher pain scores between 0 and 6 h and > 24 h. Patients with a history of opioid use/pain management had significantly higher pain scores at 6-12 h and 24-48 h. Regression analysis indicated risk factors for resistance to liposomal bupivacaine are younger patients less than 64 years old, those undergoing primary THA, and patients with a history of smoking/pain management/opioid use.

**Conclusion:**

We identify risk factors for resistance to liposomal bupivacaine, which include younger age less than 64 years old, history of smoking/pain management/opioid use. Future studies should use these risk factors as exclusion criteria when using liposomal bupivacaine or initiating any randomized trials regarding efficacy.

## Introduction

Control of postoperative pain in the setting of primary total hip and total knee arthroplasty (THA/TKA) is critical for successful outcomes leading to quicker recovery, reduced readmission rates, and lower treatment costs [1]. Currently, multi-modal pain protocols are the standard for postoperative pain control following THA/TKA [2–5]. Recently, liposomal bupivacaine gained popularity due to its extended release and initial, promising results [6]. However, not long after popularity grew, a few randomized trials reported data noting non-superior results when comparing liposomal bupivacaine to other pain modalities (within an operating multi-modal pain protocol) [7–10].

While most of the bias was removed with these level 1 trials, these studies, along with its predecessors, do not include subset analyses that may provide more insight into pain control failure [11]. In this observational regression analysis, a single-surgeon, consecutive cohort is analyzed to identify any significant risk factors for liposomal bupivacaine resistance.

## Methods

Institutional Review Board (IRB) approval, study number 20171537, was obtained to collect patient outcome data following THA and TKA at our institution. A single surgeon, consecutive cohort of 286 consecutive patients undergoing either THA or TKA between January 2016 and December 2017 were retrospectively reviewed. Retrospective analysis of institutional total joint quality and outcomes registry was screened for inclusion. Inclusion criteria consisted of any patient undergoing primary or revision arthroplasties with completed multi-modal pain management strategy and received liposomal bupivacaine (Table 1). Exclusion criteria were those undergoing any THA or TKA in the setting or past history of infection or those who were unable to receive liposomal bupivacaine (i.e. allergy).

**Table 1:** Pain control protocol for total joint arthroplasty

Preoperative	Postoperative	
	Inpatient	On discharge
Celecoxib PO - 200 mg Pregabalin PO - 50 mg (<65yo), 25 mg (>65yo) Acetaminophen IV – 1000 mg once	Celecoxib PO - 200 mg daily Pregabalin PO - 50 mg BID (<65yo) MS Contin PO – 15 mg BID (<65yo) Acetaminophen IV – 1000 mg q8h (1st 24 h) Acetaminophen PO – 650 mg q6h (> 24 h) Oxycodone PO – 5 mg q4h PRN pain 1–3 Oxycodone PO – 10 mg q4h PRN pain 4–6 Morphine IV – 2 mg q4h PRN pain 7–10 Patient-controlled analgesia^a^	Percocet PO - 5/325 mg q4-6h PRN Celecoxib PO - 200 mg daily (2 weeks) Pregabalin PO - 50 mg BID (<65yo) (2 weeks) MS Contin PO – 15 mg BID (<65yo) (2 weeks)

^a^patient-controlled anesthesia was provided at the discretion of the anesthesia department and was not offered to all patients

The standardized pain management protocol included a multi-modal approach focusing on both preoperative and postoperative pain control (Table 1). As part of a multimodal pain control regimen, liposomal bupivacaine was administered after final implants were placed prior to closure. Twenty cc of liposomal bupivacaine was diluted with 40 cc of normal saline for a total administration dose of 60 cc. Another injection is given within the surgical site consisting of 0.5% bupivacaine, morphine, and ketorolac. A 30 cc mixture of equal parts bupivacaine, morphine, and ketorolac were injected systematically into the periosteum, joint capsule, and subcutaneous tissue using an 18-guage needle. The determination of which patients would receive blocks and/or patient controlled analgesia (PCA) were at the discretion of the surgeon and anesthesiologist taking care of the patient.

As per institutional standard post-anesthesia care unit (PACU) and orthopaedic floor nursing protocol, pain scores were assessed and recorded via the Wong-Baker Visual Analog scale (VAS). Timepoints included times at 2, 4, 6, 8, 12, 24, and 48 h post-operatively.

### Statistical analysis

Frequencies and proportions of categorical patient demographic and surgical variables are reported. Means and standard deviations (SD) of continuous patient demographics and pain score variables are reported. To test for differences in demographic and surgical variables based on pain failure at 0–6, 6–12, 12–24, and 24–48 h time intervals, chi-square tests were calculated for categorical variables and one-way ANOVA tests were calculated for continuous variables. Fischer-Exact tests were used in place of chi-square tests to obtain *p*-values where > 25% of cells have expected counts < 5. The main outcome measured was highest pain score (0–10) recorded within a 48 h window post-op. The relationship between patient demographics and surgical variables with highest post-op pain score was assessed with absolute differences (AD) and 95% confidence intervals (95% CI) using bivariate and multivariable linear regression analysis. Variables with a *p*-value < 0.05 from the bivariate analysis were included in the multivariable model. A *p*-value of < 0.05 was considered to be statistically significant in all calculations. All statistical analysis was performed using SAS 9.4 (SAS Institute Inc., Cary, NC).

## Results

A total of 237 patients were included in the cohort for analysis. The average age at surgery was 62.8 years, average BMI was 32.4 kg/m^2^, and 155 (68.6%) patients were female. Surgery was performed in the right lower extremity in 133 (58.3%) patients with a primary total knee making up a majority of the procedures (54.7%), followed by primary total hip (29.6%), then revision total knee (8.1%), revision total hip (6.7%) and bilateral knee (0.9%). A majority of patients did not receive PCA (78.7%). Spinal/epidural blocks were administered to 26 (11.3%) patients, femoral/intraarticular blocks to 11 (4.8%) patients, and 193 (83.9%) received no blocks. A total of 47 (21.0%) of patients were current/former smokers, and 42 (18.7%) used alcohol. Patients with a history of pain management made up 41.8% of the population, and 42.5% of patients took pain medication at home (31% opioids, 4.0% Neurontin/Lyrica, 7.5% other). A total of 21 (9.3%) of patients were taking steroids and 23 (10.1%) taking antidepressants at the time of surgery. Diabetics made up 29.7% of the population (Table 2).

**Table 2:** Patient demographics and surgical characteristics in total population and broken down by pain failure (pain score > 5) in first 48 h post-op

	Total (*n* = 237)	Pain Failure in 0–6 h			Pain Failure in 6-12 h			Pain Failure in 12-24 h			Pain Failure in 24-48 h		
		No	Yes	*p*-value	No	Yes	*p*-value	No	Yes	*p*-value	No	Yes	*p*-value
Gender				0.37			0.30			0.86			0.71
Male	71 (31.4)	47 (33.6)	24 (27.9)		33 (29.0)	23 (36.5)		19 (33.9)	46 (32.6)		12 (28.6)	52 (31.5)	
Female	155 (68.6)	93 (66.4)	62 (72.1)		81 (71.1)	40 (63.5)		37 (66.1)	95 (67.4)		30 (71.4)	113 (68.5)	
Age	62.8 ± 10.8	64.6 ± 10.0	60.0 ± 11.5	**0.002**	63.8 ± 10.4	58.8 ± 11.8	**0.004**	64.7 ± 10.0	61.7 ± 10.9	0.08	66.5 ± 10.3	61.8 ± 10.7	**0.01**
BMI	32.4 ± 11.8	31.7 ± 6.2	33.6 ± 17.4	0.24	31.4 ± 6.6	34.2 ± 20.1	0.17	30.2 ± 6.0	33.7 ± 13.9	0.07	30.5 ± 6.5	32.8 ± 18.1	0.30
Side				0.45			0.34			0.25			0.70
Right	133 (58.3)	85 (60.3)	48 (55.2)		70 (61.4)	34 (54.0)		36 (64.3)	79 (55.2)		23 (54.8)	97 (58.1)	
Left	95 (41.7)	56 (39.7)	39 (44.8)		44 (38.6)	29 (46.0)		20 (35.7)	64 (44.8)		19 (45.2)	70 (41.9)	
Surgery				**0.03**			0.16*			0.14*			0.36*
Primary Total Knee	122 (54.7)	72 (52.6)	50 (58.1)		64 (57.7)	37 (58.7)		26 (47.3)	85 (60.7)		22 (56.4)	92 (55.4)	
Revision Total Knee	18 (8.1)	10 (7.3)	8 (9.3)		7 (6.3)	3 (4.8)		3 (5.5)	12 (8.6)		3 (7.7)	13 (7.8)	
Primary Total Hip	66 (29.6)	48 (35.0)	18 (20.9)		37 (33.3)	17 (27.0)		22 (40.0)	31 (22.1)		14 (35.9)	46 (27.7)	
Revision Total Hip	15 (6.7)	5 (3.7)	10 (11.6)		2 (1.8)	6 (9.5)		4 (7.3)	10 (7.1)		0	13 (7.8)	
Bilateral Knee	2 (0.9)	2 (1.5)	0		1 (0.9)	0		0	2 (1.4)		0	2 (1.2)	
PCA				0.36			0.86			**0.03**			0.15
Yes	49 (21.3)	109 (76.8)	72 (81.8)		90 (78.3)	50 (79.4)		38 (69.1)	120 (83.3)		31 (72.1)	137 (82.0)	
No	181 (78.7)	33 (23.2)	16 (18.2)		25 (21.7)	13 (20.6)		17 (30.9)	24 (16.7)		12 (27.9)	30 (18.0)	
Type of Block				0.42			0.76			0.37			0.68
None	193 (83.9)	117 (82.4)	76 (86.4)		96 (83.5)	55 (87.3)		42 (76.4)	122 (84.7)		37 (86.1)	137 (82.0)	
Spinal/Epidural	26 (11.3)	19 (13.4)	7 (8.0)		13 (11.3)	5 (7.9)		10 (18.2)	16 (11.1)		5 (11.6)	21 (12.6)	
Femoral/Intraarticular	11 (4.8)	6 (4.2)	5 (5.7)		6 (5.2)	3 (4.8)		3 (5.5)	6 (4.2)		1 (2.3)	9 (5.4)	
Smoker				**0.04**			0.20			0.29			**0.05**
Yes	47 (21.0)	23 (16.6)	24 (28.2)		20 (17.7)	16 (25.8)		9 (16.1)	32 (22.9)		4 (9.5)	38 (23.3)	
No	177 (79.0)	116 (83.5)	61 (71.8)		93 (82.3)	46 (74.2)		47 (83.9)	108 (77.1)		38 (90.5)	125 (76.7)	
Alcohol				0.49			0.23			0.54			0.11
Yes	42 (18.7)	24 (17.3)	18 (20.9)		17 (15.0)	14 (22.2)		9 (16.1)	28 (19.9)		4 (9.5)	33 (20.1)	
No	183 (81.3)	115 (82.7)	68 (79.1)		96 (85.0)	49 (77.8)		47 (83.9)	113 (80.1)		38 (90.5)	131 (79.9)	
Hx Pain Management				0.09			**0.0003**			0.25			**0.01**
Yes	94 (41.8)	52 (37.4)	42 (48.8)		38 (33.3)	38 (61.3)		20 (36.4)	64 (45.4)		11 (26.2)	79 (48.2)	
No	131 (58.2)	87 (62.6)	44 (51.2)		76 (66.7)	24 (38.7)		35 (63.6)	77 (54.6)		31 (73.8)	85 (51.8)	
Home Pain Meds				0.11			0.001*			0.20*			0.004*
None	130 (57.5)	86 (61.4)	44 (51.2)		76 (66.7)	24 (38.1)		34 (60.7)	77 (54.6)		31 (73.8)	84 (50.9)	
Opioids	70 (31.0)	36 (25.7)	34 (39.5)		27 (23.7)	32 (50.8)		13 (23.2)	50 (35.5)		5 (11.9)	63 (38.2)	
Neurontin/Lyrica	9 (4.0)	5 (3.6)	4 (4.7)		5 (4.4)	2 (3.2)		2 (3.6)	6 (4.3)		1 (2.4)	7 (4.2)	
Other	17 (7.5)	13 (9.3)	4 (4.7)		6 (5.3)	5 (7.9)		7 (12.5)	8 (5.7)		5 (11.9)	11 (6.7)	
Steroids				0.63			0.87			0.54			0.77*
Yes	21 (9.3)	12 (8.6)	9 (10.5)		10 (8.8)	6 (9.5)		4 (7.1)	14 (9.9)		3 (7.1)	17 (10.3)	
No	205 (90.7)	128 (91.4)	77 (89.5)		104 (91.2)	57 (90.5)		52 (92.9)	127 (90.1)		39 (92.9)	148 (89.7)	
Antidepressants				0.14			0.18			0.53			0.79*
Yes	23 (10.1)	11 (7.9)	12 (14.0)		9 (7.9)	9 (14.3)		5 (8.9)	17 (12.1)		5 (11.9)	18 (10.9)	
No	203 (89.8)	129 (92.1)	74 (86.1)		105 (92.1)	54 (85.7)		51 (91.1)	124 (87.9)		37 (88.1)	147 (89.1)	
Diabetes				0.45			0.12			0.94			0.93
Yes	67 (29.7)	44 (31.4)	23 (26.7)		40 (35.1)	15 (23.8)		16 (28.6)	41 (29.1)		13 (31.0)	50 (30.3)	
No	159 (70.4)	96 (68.6)	63 (73.3)		74 (64.9)	48 (76.2)		40 (71.4)	100 (70.9)		29 (69.1)	115 (69.7)	

*Fisher-Exact tests were used in place of chi-square tests to obtain *p*-values were > 25% of cells have expected counts < 5

Bold indicates clinical significance < 0.05

### Demographic and surgical characteristics of patients who experienced pain failure at 0-6 h, 6-12 h, 12-24 h, and 24-48 h intervals post-Op

Pain scores were measured at 0–6 h, 6–12 h, 12–24 h, and 24–48 h intervals post-op and recorded as the highest pain score within that time interval. A pain score of ≥5 was considered to be pain failure while a pain score < 5 was considered to be adequately controlled pain. A total of 91 (38.4%) patients experienced pain failure at 0–6 h post-op, 65 (35.7%) patients at 6–12 h post-op, 147 (71.4%) patients at 12–24 h post-op, and 171 (79.5%) patients at 24–48 h post-op. Mean highest pain score was 1.8 ± 1.8 in the PACU, 4.3 ± 3.1 at 0–6 h post-op, 4.2 ± 3.1 at 6–12 h post-op, 6.4 ± 2.8 at 12–24 h post-op, and 6.8 ± 2.6 at 24–48 h post-op. Overall, within the first 48 h post-op, mean highest pain score was 7.5 ± 2.2 and a total of 205 (86.5%) of patients experienced pain failure at least once (Table 3).

**Table 3:** Pain failure and highest pain score in first 48 h post-op

	Pain Failure?		Mean Highest Pain Score
	Pain Score ≤ 5	Pain Score > 5	
Pain Level			
In PACU	–	–	1.8 ± 1.8
0–6 h	146 (61.6)	91 (38.4)	4.3 ± 3.1
6–12 h	117 (64.3)	65 (35.7)	4.2 ± 3.1
12–24 h	59 (28.6)	147 (71.4)	6.4 ± 2.8
24–28 h	44 (20.5)	171 (79.5)	6.8 ± 2.6
Highest Pain 0-48 h	32 (13.5)	205 (86.5)	7.5 ± 2.2

Overall, younger patients less than 64 years old, smokers, and patients with a history of pain management had a higher rate of pain failure within the first 48 h post-op (Table 2). Patients who were younger than 64 years old at the time of surgery had a higher proportion of pain failure at 0–6 h (60.0 yrs. vs. 64.6 yrs.; *p* = 0.002), 6–12 h (63.5 yrs. vs. 71.1 yrs.; *p* = 0.004), and 24–48 h (61.8 yrs. vs. 66.5 yrs.; *p* = 0.01) intervals. Among patients with a history of pain management, a higher proportion of patients had pain failure at 6–12 h (61.3% pain score ≥ 5 vs. 33.3% pain score < 5; *p*-value = 0.0003) and 24–48 h (48.2% pain score ≥ 5 vs. 26.2% pain score < 5; *p* = value = 0.01) intervals. A higher proportion of pain failure was also observed in patients who were on opioid pain medication at home at 6–12 h (50.8% pain score ≥ 5 vs. 23.7% pain score < 5; *p*-value = 0.001) and 24–48 h (38.2% pain score ≥ 5 vs. 11.9% pain score < 5; *p*-value = 0.004) intervals. Patients who were not on any pain medication at home were less likely to experience pain failure at 6–12 h (66.7% pain score < 5 vs. 38.1% pain score ≥ 5; *p*-value = 0.001) and 24–48 h (73.8% pain score < 5 vs. 50.9% pain score ≥ 5; *p*-value = 0.004) intervals. A higher proportion of smokers experienced pain failure within the first 0-6 h postop (28.2% pain score ≥ 5 vs. 16.6% pain score < 5; *p*-value = 0.04) and patients who received PCA after surgery experienced a higher rate of pain failure 12–24 h after surgery (83.3% pain score ≥ 5 vs. 69.1% pain score < 5; *p*-value = 0.03). Gender, BMI, side of surgery, type of surgery, type of block received, alcohol usage, steroid usage, antidepressant usage, and prior diagnosis of diabetes were not significantly associated with pain failure at any time interval 0-48 h after surgery (*p*-value> 0.05) (Table 2).

### Bivariate and multivariable linear regression of highest pain score recorded within 48 h post-Op

Linear regression models were constructed with the highest pain score recorded within the first 48 h post-op as the main outcome measure (Table 4). In bivariate analysis, older patients over 64 years old had lower pain score (AD -0.05; 95% CI -0.07, 0.02) and patients with a higher BMI had a higher pain score (AD 0.03; 95% CI 0.01, 0.05). Primary THA was associated with a lower pain score compared to primary TKA (AD -0.83; 95% CI -1.48, − 0.19) and patients who received PCA after surgery had a higher pain score compared to those who did not (AD 0.70; 95% CI 0.01, 1.39). Patients who had a history of pain management had a higher pain score compared to those who had no history of pain management (AD 0.69; 95% CI 0.12, 1.27) and patients who used opioid pain medications at home had a higher pain score by 1 point compared to those who did not use any pain medications at home (AD 0.97; 95% CI 0.34, 1.59) (Table 3). Patients who underwent spinal nerve blocks were not found to have statistically significant better pain control when compared to those who had general anesthesia (AD 0.69; 95% CI -0.20, 1.59). Similar results were found when peripheral nerve blocks were compared with those patients who underwent general anesthesia (AD 0.94; 95% CI -0.39, 2.27).

**Table 4:** Bivariate and multivariable linear regression models of the highest pain score in the first 48 h post-op

	Highest Pain Score (0-48 h)			
	Bivariate		Multivariable	
	AD (95%CI)	*p*-value	AD (95% CI)	*p*-value
Gender				
Male	0	–	–	–
Female	0.29 (−0.32, 0.91)	0.35	–	–
Age	-0.05 (− 0.07, − 0.02)	0.0002	-0.05 (− 0.07, − 0.02)	0.001
BMI	0.03 (0.01, 0.05)	0.02	0.01 (− 0.01, 0.04)	0.28
Side				
Right	0	–	–	–
Left	0.22 (− 0.36, 0.80)	0.45	–	–
Surgery				
Primary Total Knee	0	–	0	–
Revision Total Knee	−0.82 (−1.89, 0.25)	0.13	−0.87 (− 1.92, 0.18)	0.12
Primary Total Hip	−0.83 (−1.48, − 0.19)	0.01	− 1.03 (− 1.68, − 0.38)	0.002
Revision Total Hip	−0.01 (− 1.17, 1.15)	0.99	− 0.23 (− 1.43, 0.98)	0.71
Bilateral Knee	2.12 (− 0.89, 5.14)	0.17	2.27 (− 0.56, 5.11)	0.12
PCA				
Yes	0.70 (0.01, 1.39)	0.05	0.40 (−0.32, 1.12)	0.28
No	0	–	0	–
Type of Block				
None	0	–	–	–
Spinal/Epidural	0.69 (−0.20, 1.59)	0.13	–	–
Femoral/Intraarticular	0.94 (−0.39, 2.27)	0.17	–	–
Smoker				
Yes	0.39 (−0.32, 1.10)	0.28	–	–
No	0	–	–	–
Alcohol				
Yes	0.28 (−0.46, 1.01)	0.46	–	–
No	0	–	–	–
Hx Pain Management				
Yes	0.69 (0.12, 1.27)	0.02	0.82 (−3.30, 4.94)	0.70
No	0	–	0	–
Home Pain Meds				
None	0	–	0	–
Opioids	0.97 (0.34, 1.59)	0.002	0.09 (−4.06, 4.24)	0.97
Neurontin/Lyrica	0.52 (−0.93, 2.00)	0.49	−0.62 (− 4.98, 3.74)	0.78
Other	−0.38 (−1.46, 0.71)	0.49	−1.07 (−5.08, 2.94)	0.60
Steroids				
Yes	0.23 (−0.76, 1.22)	0.65	–	–
No	0	–	–	–
Antidepressants				
Yes	0.50 (−0.45, 1.44)	0.30	–	–
No	0	–	–	–
Diabetes				
Yes	−0.02 (−0.65, 0.61)	0.94	–	–
No	0	–	–	–

Ref = 0

In multivariable analysis, age and primary total hip procedure were both negatively associated with higher pain scores. Older patients greater than 64 years old scored lower on pain compared to younger patients (AD -0.05; 95% CI -0.07, − 0.02) and primary total hip procedure was associated with a lower pain score by greater than 1 point compared to primary total knee procedure (AD -1.03; 95% CI -1.68, − 0.38) (Table 4).

## Discussion

Multi-modal pain protocols following THA/TKA have become the standard of care [2–5]. Oral medications, with or without the use of regional and local anesthesia make up a plethora of pain regimens, and recently liposomal bupivacaine became an important component allowing for improved pain relief for up to 72 h [12]. Several studies reported lower pain scores as the benefits of extended release bupivacaine exhibited promising results [12–16]. However, as usage grew, results of several randomized trials, made even devoted users skeptical as the data exhibited non-superior results when compared to other modalities [7–10]. As a result, many orthopaedic surgeons have moved away from use of liposomal bupivacaine citing high cost for no presumed benefit [9]. To our knowledge, however, none of these studies performed sub-cohort analyses to determine if any risk factors for liposomal bupivacaine resistance (LBR); anecdotally (and exhibited in the literature), some patients do experience a clear benefit, and this formulates the base hypothesis for this study.

Our current study investigated the efficacy of pain control after liposomal bupivacaine injection following TKA/THA at various time intervals in order to ascertain risk factors leading to resistance of liposomal bupivacaine. Results from our study demonstrated that a total of 86% of the 237 patients included had pain failure at least once during the first 48 h. Not surprisingly, smokers, younger patients less than 64 years old, and patients with prior opioid use, experienced significantly higher rates of pain failure at various time intervals (Table 2) [17–20]. Previous literature has demonstrated that older patients tend to use less opioids than younger patients which may result from changes in metabolism and clearance of opioid drugs with increased age [17–19]. Recent literature on how age influences post operative pain following TKA or THA is contradictory and has demonstrated no difference in pain in some studies, while others show younger patients have more pain [21, 22]. In addition, prior studies have demonstrated that smoking has been associated with high pain scores when compared with non-smoker counterparts [20]. Smoking has shown associated with changes in levels of neuropeptides that play a role in chronic pain, and patients who smoke typically have lower plasma beta-endorphin levels [23, 24]. Lastly, the induction of cytochrome P-450 isoenzymes by tobacco smoke can increase the metabolism of opioids thus leading to inadequate pain control [25, 26].

This study does have some limitations. Power calculation at 0.80 and alpha of 0.05, yielded a R-squared coefficient of 87.5% indicating significance for the regression analysis performed. At a power of 0.90, however, R-squared coefficient fell to 52.3%, offering a less reliable significance at a higher power. However, with a significant R-squared at a power of 0.80, the authors considered the statistical significance appropriately interpreted for the conclusions found in this study. Another limitation of this study is a lack of control group. When examining our data, we found that our baseline pain scores were in line with historical controls; therefore, a control group was assessed. In addition, retrospective analysis of a registry may have led to potential inherent bias; however, the goal of this study was not to demonstrate superiority but rather identify risk factors for failure of pain management with liposomal bupivacaine. Lastly, there were select patients who were provided with PCA, regional block, or spinal anesthesia, which may influence pain scores for patients receiving liposomal bupivacaine, but this was also taken into consideration in our statistical analysis.

## Conclusion

Liposomal bupivacaine has been shown to be efficacious in pain control management following primary and revision arthroplasties. However, our study demonstrated that younger age less than 64, and a history of smoking, pain management, and opioid use, led to failed pain management after use of liposomal bupivacaine as part of a multi-modal pain regimen. Future studies should use these criteria as exclusion criteria when using liposomal bupivacaine or initiating any randomized trials regarding efficacy.

## Data Availability

The datasets used and/or analyzed during the current study are available from the corresponding author on reasonable request.

## Abbreviations

AD: Absolute Difference
BMI: Body Mass Index
CI: Confidence Interval
IRB: Institutional Review Board
LBR: Liposomal Bupivacaine Resistance
PACU: Post-Anesthesia Care Unit
PCA: Patient Controlled Analgesia
SD: Standard Deviation
THA: Total Hip Arthroplasty
TKA: Total Knee Arthroplasty
VAS: Visual Analogue Scale
